# Periodotopy in the gerbil inferior colliculus: local clustering rather than a gradient map

**DOI:** 10.3389/fncir.2015.00037

**Published:** 2015-08-04

**Authors:** Jan W. H. Schnupp, Jose A. Garcia-Lazaro, Nicholas A. Lesica

**Affiliations:** ^1^Department of Physiology, Anatomy and Genetics, University of OxfordOxford, UK; ^2^Ear Institute, University College LondonLondon, UK

**Keywords:** inferior colliculus, tonotopy, periodotopy, periodic sound, pitch, auditory midbrain, functional anatomy

## Abstract

Periodicities in sound waveforms are widespread, and shape important perceptual attributes of sound including rhythm and pitch. Previous studies have indicated that, in the inferior colliculus (IC), a key processing stage in the auditory midbrain, neurons tuned to different periodicities might be arranged along a periodotopic axis which runs approximately orthogonal to the tonotopic axis. Here we map out the topography of frequency and periodicity tuning in the IC of gerbils in unprecedented detail, using pure tones and different periodic sounds, including click trains, sinusoidally amplitude modulated (SAM) noise and iterated rippled noise. We found that while the tonotopic map exhibited a clear and highly reproducible gradient across all animals, periodotopic maps varied greatly across different types of periodic sound and from animal to animal. Furthermore, periodotopic gradients typically explained only about 10% of the variance in modulation tuning between recording sites. However, there was a strong local clustering of periodicity tuning at a spatial scale of *ca*. 0.5 mm, which also differed from animal to animal.

## Introduction

Many natural sounds are periodic. Inanimate resonators easily enter into periodic oscillations at frequencies which are revealing about their physical size and weight, and animate sound sources produce a plethora of periodic sounds, from wing beats to vocalizations. Humans perceive periodicities at rates below 30 Hz as rhythmic, and those above 30 Hz as a tonal buzz. Thus, the qualities of rhythm and pitch both derive from processing periodicity in acoustic waveforms. Detecting and processing periodicity in sounds is clearly a key task for the auditory system that can be achieved in a number of different ways (Frisina, 2001; Joris et al., 2004; Schnupp and Bizley, 2010; Schnupp et al., 2011). The periodicity of a sound may be evident in the time domain as repetitions of a waveform motif, or in the frequency domain as spectral peaks at harmonics of a fundamental frequency F0, where F0 is the inverse of the sound’s period. The spectral cues are thought to be encoded by a place code, carried by the pattern of activity across the tonotopic nerve fibers in the lemniscal auditory pathway, while time domain cues manifest as periodic amplitude modulations (AM) of the stimulus envelope, which are encoded when neurons in the auditory periphery “phase-lock” their discharge patterns to the stimulus modulations. Early stages of the mammalian auditory system are thought to use both temporal and spectral cues to varying extent, with increasingly higher stages of the auditory pathway relying less on temporal discharge patterns and more on the overall discharge rates to represent stimulus periodicity (Frisina, 2001; Joris et al., 2004). One popular interpretation of the apparent dependence of firing rates on the modulation rates of periodic stimuli in midbrain and cortical neurons is that the auditory system may implement a “modulation filter bank” to process incoming sounds, and conceptual models of the auditory pathway based on this hypothesis (Jepsen et al., 2008) have been able to generate plausible explanations for a variety of psychoacoustic phenomena.

Work by Schreiner and Langner suggested that, in the inferior colliculus (IC) of the cat, neurons are tuned to different preferred periodicities and may form a periodotopic map (Schreiner and Langner, 1988). This idea is attractive: a periodotopic map could, for example, physically separate simultaneous sounds which differ in pitch. More recent functional magnetic resonance imaging (fMRI) experiments by Baumann et al. (2011) further support the idea that the IC may exhibit a periodotopic gradient. However, because of the difficulty in collating results across single electrode recordings and the coarse spatial and temporal resolution of fMRI, the results of these previous studies are not definitive, and it is worth noting that a study by Müller-Preuss et al. (1994) failed to confirm the existence of a periodotopic map. Furthermore, these previous studies relied on responses to a single type of periodic sound, so the robustness of the topographic structure of periodicity tuning to changes in other acoustic features has not yet been tested. In this study, we presented three different types of periodic sounds to gerbils and used array electrodes to record responses across three dimensions of the IC in individual animals with a spatial resolution of 0.2 mm or better. We find that neural periodicity tuning depends strongly on stimulus type and, in contrast to previous studies, is not strongly constrained by periodotopic gradients; instead, strong local clustering of neurons with similar periodicity tuning results in a topography similar to the well-known orientation maps in the visual cortex of higher mammals.

## Materials and Methods

### *In vivo* Extracellular Recordings

All procedures on animals were approved and licensed by the University College of London, London, UK (UCL) Animal Welfare and Ethical Review Body (AWERB) as well as the UK home office in accordance with governing legislation (ASPA 1986). Experiments were conducted in a sound-insulated chamber (Industrial Acoustics, Winchester, UK). Adult male Mongolian gerbils (*Meriones unguiculatus*) with typical weights between 70–90 g and ages ranging between 2–4 months were used in this study. Anesthesia was induced by intraperitoneal injection of 0.65 ml per 100 g body weight of a mixture of five parts of ketamine (100 mg/ml), one part of xylazine (20 mg/ml), and 19 parts of physiological saline. To maintain anesthesia, the same solution was infused continuously during recording at a rate of approximately 2.1 μl/min. Body temperature was maintained at 38.7°C by a heating blanket that was controlled via feedback from a rectal probe. The skull was exposed by incision of the scalp and a metallic pin was cemented to it. Subsequently the pin was coupled to a stainless steel head holder in a stereotaxic frame.

A craniotomy was performed on the right side of the skull extending 3.5 mm from the mid-line and centered along the lambdoid suture. The dura was removed and the IC was exposed by aspiration of the overlying cortex and removal of the protrusion of the temporal bone which partly covers the IC in this species. Oxygen-enriched air was delivered to the vicinity of the snout and ECG and body core temperature were monitored throughout the duration of the experiment.

To map the responses throughout the extent of the IC, we used 64-channel electrode arrays (Neuronexus Technologies, Ann Arbor, MI, USA) with 175 μm^2^ recording sites arranged in a square grid pattern at 0.2 mm intervals along eight shanks with eight channels per shank. Electrodes were inserted into the IC under visual guidance and advanced slowly in 5 μm steps using a micromanipulator (Scientifica, Uckfield, East Sussex, UK). In some electrode penetrations, the array was advanced by 0.1 mm after a first set of stimuli were recorded, to achieve finer spatial sampling than a single placement of a 0.2 mm spaced grid electrode would allow. The sampling rate for the recordings was 24 kHz.

### Quantifying Neural Activity

**Figure 1:**
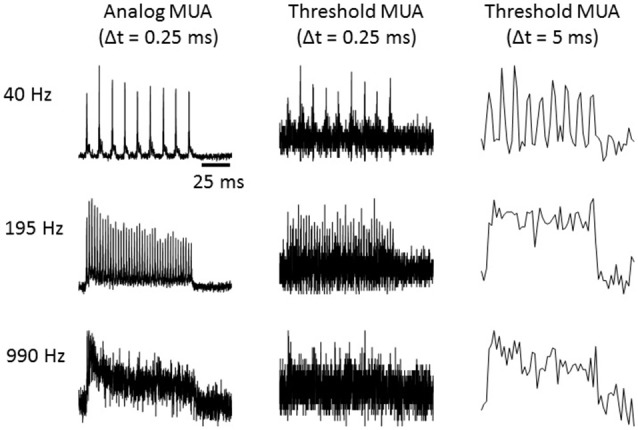
**Multi-unit responses quantified using either the “analog multi-unit activity (MUA)” method, or by counting threshold crossings with time bins as indicated**. Responses to click trains presented at the click rates indicated to the left are shown. Each trace is the average response over 40 trials.

We quantified multi-unit activity (MUA) using a measure related to the voltage signal power in the frequency band occupied by extracellularly recorded action potentials. Specifically, MUA was measured by extracting the envelope of the band pass filtered voltage recordings as follows: (1) a band pass filter was applied between 300 and 6000 Hz; (2) the absolute value was taken; and (3) a low pass filter was applied below 6000 Hz (to avoid aliasing) and the signal was downsampled to 12 kHz. A number of previous studies (Chung et al., 1987; King and Carlile, 1994; Schroeder et al., 1998; Kayser et al., 2007; Choi et al., 2010) have used related or identical methods to derive an “analog measure of MUA” (aMUA) by band-passing electrode signals (either in the frequency or time domain) to quantify neural activity by measuring signal amplitude or power in the frequency range covered by spikes. This approach is preferable to MUA measurements based on thresholding and event counts, as it does not require the choice of any free parameters and provides a substantially less noisy measure when compared to thresholding. Consider that electrical noise will on some occasions interfere with some MUA spikes to push their amplitudes below the threshold, which will cause false negatives, while on other occasions noise events will sum to exceed threshold, leading to false positives, and on yet other occasions several MUA spikes will collide in time leading to only a single threshold crossing and undercounting. Thresholding involves rounding to the nearest binary value, which increases the error in the signal in a manner analogous to the digitization noise which plagues signals that are sampled with an insufficient bit depth. The noisiness of the threshold MUA is often dealt with pragmatically by binning threshold crossing in relatively large time bins, which reduces noise by averaging over time but precludes the study of locking to high modulation rates. Figure 1 illustrates the much higher signal quality obtained at a 0.25 ms bin size with the aMUA measure compared to a threshold MUA calculated by thresholding the highpass filtered electrode signal at two standard deviations of the fluctuations in spontaneous activity and binning threshold crossings with either 0.25 ms or 5 ms bins. Results are shown for click train stimuli at three different click rates. The improved ability of aMUA to reveal time locking of the neural responses to the temporal structure of the stimulus at high modulation rates is readily apparent.

### Histology

At the end of the recording experiments the animals were overdosed with sodium pentobarbital, their heads were removed and left submerged in a fixative of four percent paraformaldehyde dissolved in phosphate buffered saline for a period of at least 1 week. After this initial fixation, the brains were extracted and cryoprotected by immersion in 30% sucrose dissolved in the paraformaldehyde fixative for a further 2 days. The cryoprotected midbrains were sliced in 50 μm coronal sections using a freezing microtome and Nissl stained to facilitate reconstruction of the electrode tracks. We used a combination of stereotaxic information gathered during electrode placement, anatomical observations from post mortem brains and from published sources (Cant and Benson, 2005), and physiological response criteria to reconstruct and register the electrode grid locations relative to each other and relative to the boundaries of the central nucleus of the IC (ICc).

### Sound Delivery Hardware

Custom earphones made from Panasonic RPHV27 headphone drivers (Bracknell, UK) and coupled to otoscope specula were inserted into each ear canal to deliver sound stimuli diotically. The headphones were calibrated to have a flat frequency output up to 24 kHz (correct to within ± 3 dB). This was achieved by connecting the earphones to an “artificial ear” comprising a 1/8″ microphone (model 40DD, G.R.A.S. Sound and Vibration, Denmark) to mimic the ear drum and a piece of silicone rubber tubing (i.d. 3.2 mm, distance microphone to tip of headphone speculum 7 mm) mimicking the ear canal. The “headphone-to-artificial-eardrum” transfer function recorded with this device was then compensated for by convolving stimuli with an inverse filter. The “artificial ear” measurements were also used to determine the absolute sound levels of the stimuli used.

### Assessing Pure Tone Frequency Tuning

Frequency tuning at each recording site was assessed by collecting traditional frequency response areas (FRAs) using 75 ms pure tone pips (2 ms cosine onset and offset ramps) repeated every 150 ms, with frequencies chosen from 256 Hz to 37.641 kHz in 1/5 octave steps, and sound levels between 10 and 80 dB SPL. Sounds of different frequencies and intensities were randomly interleaved, and five responses were collected for each frequency/intensity combination. The best frequency (BF) for each neuron was determined to be the tone frequency that produced the largest response averaged over the five highest sound levels.

### Assessing Tuning to Stimulus Periodicity

To assess tuning for stimulus modulation we used three different types of stimuli which are widely used in physiological studies of neural tuning for periodicity or modulation redundant “tuning”: click trains, sinusoidally amplitude modulated noises (SAMNs) and iterated rippled noises (IRNs).

To motivate the choice of the three different stimulus types and place them in the context of previous work, it is worth pointing out that the use of the terms “stimulus periodicity” and “periodotopy” in the published literature is not unambiguous.

Many natural stimuli, including the large majority of animal communication sounds, have highly periodic waveforms, and this periodicity manifests both in the fact that the waveforms of subsequent stimulus periods are very similar (which constrains their spectra to have a harmonic structure), as well as in envelope AM which are in step with the fundamental period. Thus, for many natural periodic sounds, the “modulation frequency” (MF) and the fundamental frequency (F0) are the same. In contrast, SAM noise, which is popular in laboratory studies, exhibits strong AM, but it is not periodic in the strict sense since the noise tokens in subsequent periods are uncorrelated. Consequently SAM noise has a well-defined MF, but its F0 is poorly defined. Similarly, SAM tones are only strictly periodic when the carrier frequency is an integer multiple of the modulator frequency. If not, successive periods are out of phase with each other, and the stimulus F0 is again undefined or ambiguous. Periodic modulation and periodicity are thus not quite the same thing, and it is in principle possible for neurons to exhibit “AM tuning” which is different from their “periodicity tuning”. This distinction is not always clear in previous studies; in fact, the two studies that provide the strongest evidence for “periodotopic” organization in the IC have used exclusively SAM tones (Schreiner and Langner, 1988) or SAM noise (Baumann et al., 2011), stimuli which exhibit periodic modulations, but are not strictly periodic.

**Figure 2:**
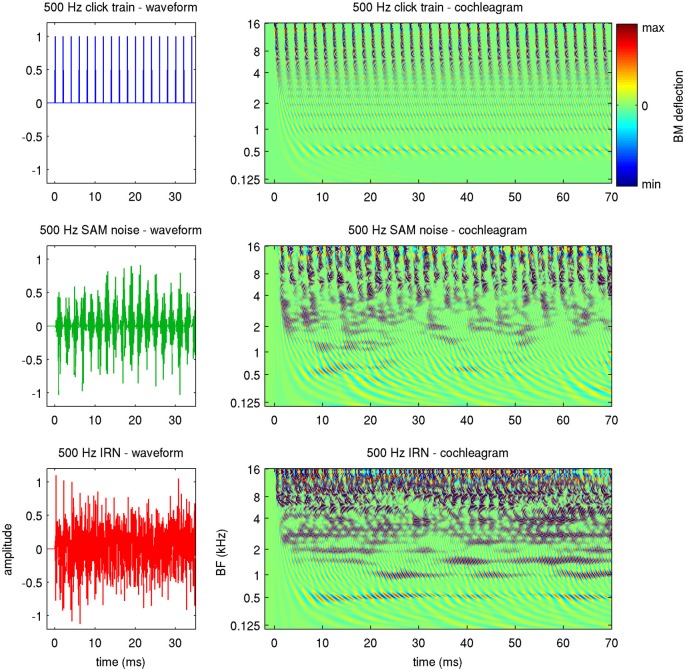
**Waveforms (left) and cochleagrams (right) of the three types of periodic stimuli used in this study**. The cochleagrams were calculated by passing the stimulus waveforms through a gammatone filter bank, and provide an estimate of the basilar membrane (BM) deflection expected along the tonotopic array (*y*-axis) as a function of time (*x*-axis).

To avoid such potential ambiguity, we shall refer to a stimulus’ “envelope periodicity” to describe any regular AM it may have, and shall use the term “fine structure periodicity” to describe the degree to which the waveforms in successive periods are correlated. The three stimulus types used in this study effectively probe these different types of “stimulus periodicity” to varying extent. Click trains are perfectly periodic, both in envelope and fine structure. SAMN has strong envelope periodicity but little or no fine structure periodicity, while for IRNs, the repeated delay and add operation used in their creation ensures a high degree of fine structure periodicity, but their envelope periodicity is much weaker. Figure 2 illustrates this, showing the first 35 ms of the stimulus waveforms, as well as the first 70 ms in a “cochleagram” representation, for stimuli with a stimulus period of 2 ms, or, equivalently, a MF of 50 Hz.

The cochleagrams were generated by passing the stimuli through a gammatone filter bank to approximate the pattern of excitation of the basilar membrane (BM) that these stimuli produce (Patterson et al., 1995). Thus, they illustrate how these stimuli will appear to the tonotopic array of auditory nerve fibers. The fine structure periodicity of the click trains causes them to have a precise harmonic structure, which manifests in the cochleagram as a series of horizontal stripes at the harmonics of the stimulus period (0.5, 1, 1.5 and 2 kHz in the example shown). At the same time, the envelope periodicity of the click train leads to “envelope phase-locking” which is synchronized across frequencies and is manifest as a series of vertical bands, spaced at the stimulus period, in the higher frequency channels. The cochleagram for the SAMN also exhibits the periodic vertical bands in the upper frequency channels, but the horizontal stripes at the lower harmonics which would be diagnostic of fine structure periodicity are much less clear. In contrast, the cochleagram of the IRN features clearer horizontal bands (i.e., a clearer harmonic structure) than the SAMN, but since the frequency components of this stimulus are not in phase with each other, IRN exhibits only weak envelope modulation, and little synchronized envelope phase-locking in the upper frequency channels.

The stimuli were generated in 200 ms long sound bursts, with 25 different MFs, from 15–4000 Hz (an interval of > 8 octaves), spaced at approximately 1/3 octave intervals, but rounded to ensure an integer number of cycles in the 200 ms stimulus duration. Hence MF ∈ {15, 20, 25, 30, 40, 50, 60, 75, 95, 120, 155, 195, 245, 310, 390, 490, 620, 785, 990, 1250, 1575, 1990, 2510, 3170, 4000} Hz. Click trains were generated as regular trains of impulses (Kronecker delta functions) spaced at intervals corresponding to 1/MF. SAM noise was generated by multiplying a Gaussian noise carrier with an envelope function of the form *y* = −0.5 *cos(2π MF t)+0.5*. IRN was generated by summing six time delayed copies of Gaussian white noise, where the *N*^th^ copy of the noise was time shifted by a delay of *(N-1)/MF*. For each stimulus class we recorded 64 responses to each of the 25 different values for MF, presented in a randomly interleaved manner, at rates of 2.5 stimuli/s and sound levels of ca. 74 dB SPL.

In this study we looked both at overall activity measures of modulation tuning as well as at temporal synchronization of neural responses to the stimulus periodicity. We shall refer to the dependence of mean response strength on MF as “response modulation transfer function” (rMTF). The “best modulation frequency” (BMF) at a particular recording site was defined simply as that MF at which the rMTF reached its maximum. The tendency of a neuron to respond to periodic stimuli with periodic firing was quantified by calculating the mean correlation coefficient of response patterns in subsequent stimulus cycles. If neural responses time lock reproducibly to individual stimulus cycles, then the discharge patterns observed in subsequent cycles will be similar and their correlation will be high. We refer to the function of the correlation coefficient for subsequent cycles as the temporal modulation transfer function (tMTF).

## Results

**Figure 3:**
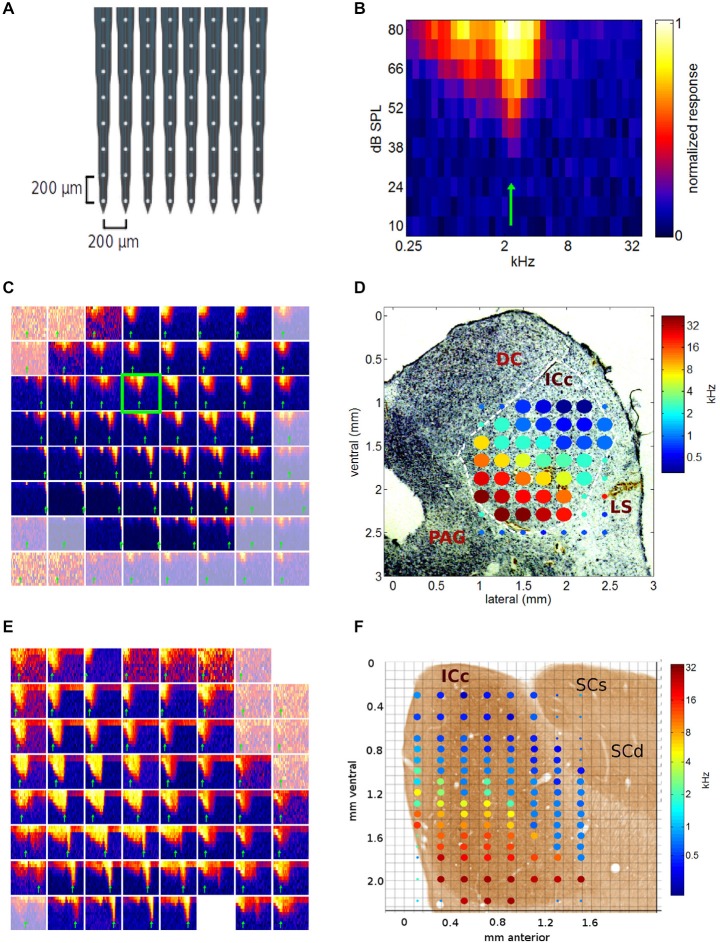
**(A)** Schematic diagram of the electrode array used in this study. **(B)** Example of a typical multiunit frequency response area (FRA) recorded in ICc. The blue-to-yellow color scale indicates the multiunit response strength observed for pure tones presented at the frequency and intensity indicated on the *x*- and *y*-axis respectively. The best frequency (BF) indicated by the green arrow was measured by summing responses for stimulus amplitudes >50 dB SPL. **(C)** FRAs collected during one medio-lateral multi-electrode array penetration through the inferior colliculus (IC). Each of the 64 squares is one FRA, with *x*- and *y*-axis ranges as in **(B)**. Channel 27 is highlighted by a green frame, as it is the same data as that shown in more detail in **(B)**. FRAs from recording sites outside the ICc are shown in a desaturated color map. **(D)** BFs derived from the data shown in **(C)** plotted as a color-coded tonotopic map superimposed to scale, onto a Nissl stained histological section through the corresponding part of the IC. The color of the filled circles indicates the BF for each recording site according to the color scale on the right. Large filled circles show BFs of recording sites located within ICc, small circles indicate recording sites outside ICc. The axes show coordinates in mm from the mid-line and the dorsal edge of the midbrain respectively. DC, dorsal cortex of the IC; LS, lateral shell of the IC; PAG, periaqueducal gray. **(E)** FRAs as in **(C)**, recorded during a multielectrode penetration oriented along the sagittal plane. Two of the channels on this multielectrode array were faulty, hence two FRAs are missing, one at the top right, the other in the bottom row. **(F)** Composite showing the BFs obtained from the data shown in **(E)**, as well as the data from a second recording location, 0.5 mm deeper, superimposed to scale onto a photo-micrograph of a sagital section through the gerbil IC taken from page 21 of Cant and Benson (2005; by kind permission of the publisher). SCs, superior colliculus, superficial layers; SCd, superior colliculus, deep layers.

We recorded multi-unit responses to pure tones and periodic stimuli from 24 placements of a planar 64-channel electrode array (see Figure 3A) in the IC of a total of six adult gerbils. Twelve of these placements were oriented medio-laterally, and the other 12 were oriented rostro-caudally. Some arrays had a small number of faulty channels, and some channels along the edges of the arrays came to lie outside the bounds of the central nucleus of the IC (Cant and Benson, 2005). A total of 1022 recording sites from the 24 multi-electrode placements were found to be in the ICc.

### Visualizing Tonotopic Gradients

**Figure 4:**
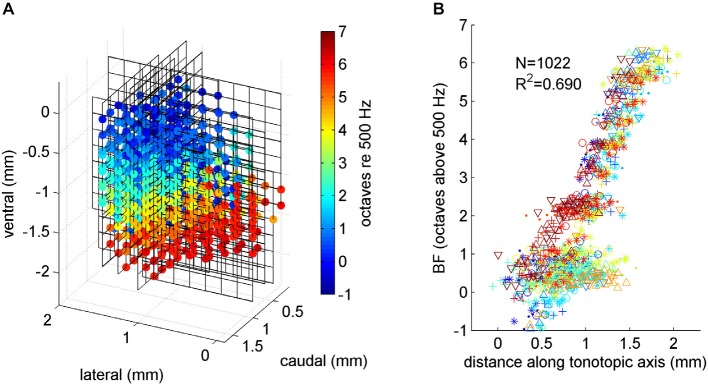
**(A)** Tonotopy in ICc. Each 8 × 8 line grid shows the positioning of one 64 channel array electrode placement, relative to the top, medial and rostral edge of the ICc. Each colored dot shows an electrode recording site inside the ICc. The color of the dot indicates the BF recorded at the corresponding location. A systematic progression from low BFs (blue colors) near the top to high BFs (red colors) near the bottom is readily apparent. These data were subjected to linear regression analysis to determine the best fit tonotopic axis of the gerbil ICc. **(B)** Multiunit BF plotted against location along the tonotopic axis. Data from each of the six animals in our study were plotted using a different symbol and color. A random Gaussian distributed jitter with a standard deviation of 0.09 octaves has been added to the BF values to spread out overlapping data points.

A long appreciated characteristic of ICc neurons is that, when probed with pure tone stimuli, they normally exhibit clear frequency tuning, and their best frequencies follow a well-defined tonotopic gradient (Hind et al., 1963; Clopton et al., 1974; Merzenich and Reid, 1974; Ryan et al., 1982; Schreiner and Langner, 1997; Malmierca et al., 2008). Documenting this tonotopic gradient in detail in our data was important to validate our methodology; the results of this analysis are shown in Figures 3, 4. Figure 3B gives a representative example of a FRA recorded at one recording site. To determine each multiunit’s BF, we averaged the responses for each frequency across all sound levels above 50 dB SPL. The frequency corresponding to the peak of this tuning curve (shown by the green arrow) served as estimate of the multiunit’s BF. For completeness, multiunit “characteristic frequencies” (CFs), that is, those pure tone frequencies that exhibited the lowest response thresholds, were also analyzed.

We shall present pure tone data from a few sample multielectrode penetrations in Figure 3 and then assemble these to form a comprehensive 3-D tonotopic map of the nucleus in Figure 4. The tonotopic organization ran in a clear dorso-ventral to medio-lateral direction, as can be seen in Figure 3C, which shows all 64 FRAs recorded during a single, medio-laterally oriented multielectrode penetration. The FRA shown in more detail in Figure 3B is framed in green in Figure 3C. Recording sites lying beyond the edges of the ICc are shown using a desaturated color scale. It is readily apparent that FRAs recorded within ICc at the top right of the grid of recording sites tend to be tuned to relatively low frequencies, while FRAs in lower rows and further to the left tend to be tuned to progressively higher frequencies. In other words, multiunit BFs follow a tonotopic gradient which runs from the dorsal and lateral edge of the ICc in a medio-ventral direction. This is particularly clearly visible in Figure 3D, which shows the BF values extracted from the FRAs shown in Figure 3C projected to scale onto a photo-micrograph of the corresponding Nissl stained section of the midbrain. Note that pure tone thresholds were generally lowest near the center of the ICc, as would be expected given the general shape of the audiogram, and in the light of previous studies of the rodent IC (Stiebler and Ehret, 1985; Stiebler, 1986).

Figure 3E shows further examples of FRAs, this time obtained from a rostro-caudally oriented multielectrode penetration. A tonotopic gradient is again readily apparent, with more ventral recording sites exhibiting higher BFs than dorsal ones. Figure 3F shows the BFs obtained from the data shown in Figure 3E, as well as the data from a second location with the same medio-lateral and rostro-caudal coordinates but 0.5 mm deeper, superimposed onto a photo-micrograph of a corresponding sagittal section of the IC. Our own histological sections were all cut along the coronal plane, therefore, the composite shown in Figure 3F incorporates a histological image taken from the atlas of the gerbil IC by Cant and Benson (2005).

To reveal the tonotopic organization of the ICc across all three spatial dimensions we show in Figure 4A the BF data, compiled and brought into register from all 24 multielectode placements, and projected onto a 3-D coordinate system shown in perspective. The coordinate system is in mm relative to the medial edge (*x*-axis), the rostral pole (*y*-axis) and the dorsal surface (*z*-axis) of the ICc. The line grids show the relative positions of the electrode grid placements. The grid vertices show the recording sites, and those which were positioned inside the ICc are marked with colored dots, with the color indicating each site’s BF. The progression of low BFs (blue colors) at dorso-lateral through mid-range BFs (greens and yellows) toward high BFs (red colors) at ventro-medial locations is clear and striking, and in excellent agreement with previous reports of tonotopy in the IC.

### The Tonotopic Organization is Well Described by a Linear Tonotopic Gradient

To model the tonotopy quantitatively, we fitted a linear regression of BF against the multiunits’ anatomical coordinates along the medio-lateral, rostro-caudal and ventro-dorsal axis. Since we would expect the tonotopic axis to represent BFs on a logarithmic, octave scale rather than on a linear frequency scale, we converted BF values to octaves relative to 500 Hz.

Regressing BF in octaves against anatomical location yielded the regression equation:

(1) $$\hat{B}F=-2.03\cdot x+1.78\cdot y+3.07\cdot z+0.053$$

where $\hat{B}F$ is the estimated BF in octaves, *x* is the coordinate lateral from the medial edge of the central nucleus in mm, *y* is the distance caudal from its rostral pole, and *z* is the distance ventral from the dorsal pole. This regression can account for 69% of the observed variance in BFs, and it results in a simple model which facilitates the calculation of various useful descriptive summary statistics. For example, the regression weights form a vector *u* = [−2.03, 1.78, 3.07] which defines the tonotopic axis. If we move along the tonotopic gradient in the direction of *u* by 1 mm, we would expect BFs to increase by ||*u*|| = 4.08 octaves. Projected onto a frontal section, the tonotopic axis runs lateral to medial at an angle of 56.6° relative to the horizontal. Seen in the sagittal plane, the tonotopic axis runs rostral to caudal at an angle of 59.9° relative to the horizontal.

For completeness we repeated the above analysis using CF instead of BF. We found BF and CF to be highly correlated, and obtained almost identical results for the topographic analysis with CF. The best fit tonotopic axes for CF were aligned with those for BF to about 6°, but the scatter around the best fit tonotopic axis was slightly higher for CF than for BF (data not shown). We attribute this increased scatter to the fact that CF estimation is inherently less precise than that of BF, given that, by design, CF estimation tries to detect near threshold responses very close to the neural “noise floor”, while BF estimates are based on quantifying the strength of robust responses to stimuli well above threshold. We shall focus on reporting BFs for the remainder of our analysis.

This linear tonotopic gradient model of BFs in ICc is not only appealingly simple, it also fits the data from all animals in our sample very well, as can be seen in Figure 4B, which plots multiunit BF against anatomical location measured along the tonotopic axis revealed by the regression analysis. In Figure 4B, different colors and symbols are used to distinguish data from each of the six animals. It illustrates that BFs from all the animals are all well accounted for by the regression model given by Eqn. (1). In other words, the tonotopy is highly conserved from one animal to the next, and particularly for BFs >1 kHz, modeling tonotopy as a simple linear regression on anatomical coordinates accounts for observed BFs very well. Multiunits with BFs <1 kHz are, interestingly, more widely scattered anatomically than the simple linear regression model would predict. This finding is in agreement with anatomical studies noting the absence of a clear laminar structure in the dorsal part of the gerbil ICc (Cant, 2013). The fact that our experimental approach reveals the tonotopic organization of ICc in great clarity and detail confirms its suitability for the study of the topography of neural feature selectivity.

### Modulation Tuning Curves are Very Diverse and Differ for Different Stimulus Types

**Figure 5:**
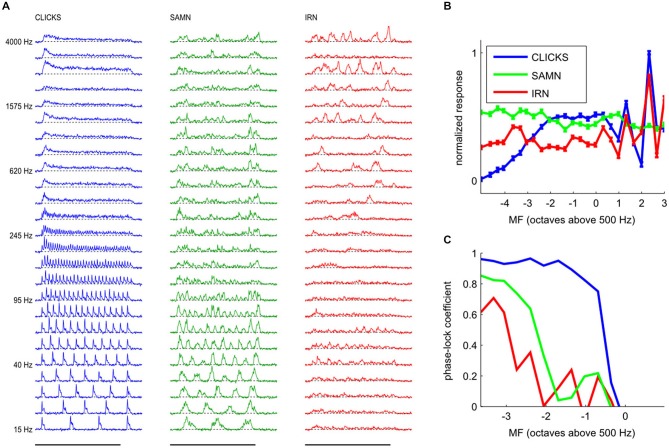
**(A)** Examples of responses from a typical multiunit with a pure tone BF of 7.49 kHz to click trains (left, blue), SAMN (middle, green) and IRN (right, red) for the full range of fundamental frequencies tested. Each line shows the median response amplitude over 25 presentations of the stimulus with modulation frequency (MF) as shown on the left. The x-coordinate represents post-stimulus time, with the black bars at the bottom indicating the duration of stimulus presentation (200 ms). **(B)** Rate modulation transfer functions (rMTFs) derived from the data shown in **(A)** by plotting the total response (area under the curves in **A**) as a function of stimulus MF. Error bars show standard error of the mean. Standard errors were small, so error bars may be hard to see. It is evident that both the shape and the maxima of the rMTF were strongly stimulus dependent; the rBMFs for this multiunit were 2510 kHz for click trains and IRNs, but only 95 Hz for SAMN. **(C)** Temporal modulation transfer functions (tMTFs), calculated as described in the “Materials and Methods” Section, for the responses shown in **(A)**.

Having verified that our methodology can reveal anatomical gradients in physiological response properties with great clarity, we next examined responses to periodic stimuli. As briefly mentioned in the introduction, neural coding for periodic stimuli can be quantified in a number of different ways. Commonly used approaches are based either on rate-based measures of the dependence of mean evoked response strength on stimulus MF (Schreiner and Langner, 1988; Miller et al., 2002; Baumann et al., 2011), or they quantify the extent to which neural discharges are time-locked to the stimulus period, using vector strength calculations or autocorrelation methods (Frisina et al., 1990, 1996; Wiegrebe and Winter, 2001) or both (Rees and Palmer, 1989). Either of these approaches can, in principle, serve to investigate responses to periodic stimuli in the ICc, but they would not necessarily yield the same answers. To illustrate this, consider the example of responses from a typical multiunit to click trains, SAMN and IRN shown in Figure 5A.

In this example, it is clear that all three types of periodic stimuli were able to produce sizable responses over wide ranges of MFs, and that the responses (and hence the nature and extent of “tuning” for stimulus MF) were quite different for each of the three periodic stimulus classes used in this study. At this recording site, click trains evoked the strongest responses, and click trains with MFs up to about 250 Hz evoked periodic peaks of activity, while click trains with MFs substantially above 250 Hz evoked sustained activity which can vary greatly in amplitude depending on MF. Note, for example, that the total response amplitude (area under the blue curves) for MF = 2510 Hz (third row from the top) is much larger than that seen in response to MFs just above or below 2510 Hz. When analyzed using an “overall rate” response measure, the multiunit’s MF tuning curve for click trains therefore exhibits several pronounced peaks, some at MFs well above 1000 Hz, as can be seen in Figure 5B, which shows the multiunit’s MF tuning curve (or rMTF) as a blue line.

It is also readily apparent that this multiunit phase-locked strongly to the stimulus period for click trains at low modulation rates, as responses in each stimulus cycle exhibit highly reproducible patterns, but this ability to phase lock declines at higher modulation rates. We quantified phase locking by calculating the mean correlation of the response patterns in successive periods of the stimulus as a function of MF. The resulting tMTF is shown in Figure 5C, and it is “low pass” with a cutoff between approximately 220 and 440 Hz.

In comparison, phase-locking to SAMN was somewhat less regular and less pronounced than that to click trains, and also declined at lower MFs than for click trains. At MFs above the phase-locking cutoff, the responses to SAMN appear more irregular and less sustained than the responses to click trains. In comparison, when tested with IRNs at low MF, this particular multiunit responds only relatively weakly, and phase-locks only weakly at MF around 60 or 75 Hz (compare Figure 5C) while IRN stimuli with high MFs can evoke strong but irregular responses. Thus, for all three stimulus types shown, stimulus MF greatly influences the evoked response, but the type of “periodicity tuning” one observes for this multiunit depends on stimulus type and whether one chooses rate- or timing-based measures. Interestingly, this multiunit’s rMTF for IRN and for click trains exhibited similar sharp peaks at higher MFs, but the rMTF for SAMN was relatively flat without such features. Considering that, as discussed in the context of Figure 2 in the “Materials and Methods” Section, IRN has mostly “fine structure periodicity”, SAMN has mostly “envelope periodicity”, and click trains have both, this observation hints at the possibility that this multiunit might be more sensitive to fine structure than envelope periodicity.

**Figure 6:**
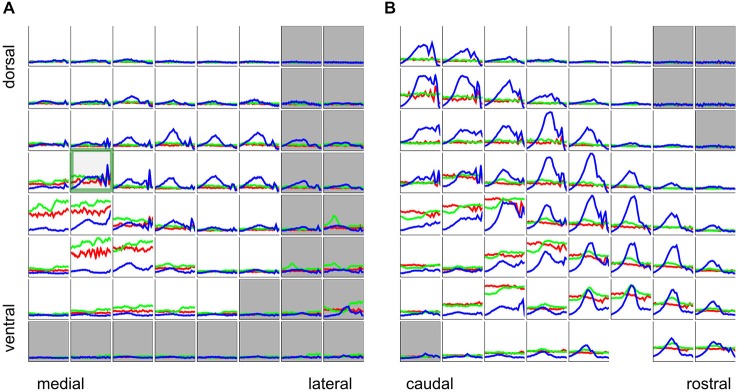
**Rate tuning to periodicity in click trains (blue), SAMN (green), and IRN (red) for two sample electrode penetrations, one oriented medio-laterally (A) the other oriented rostro-caudally (B)**. Each small square shows the responses for the corresponding recording site in the 8 × 8 electrode array. Each colored curve gives a tuning curve, with stimulus MF on the *x*-axis and response amplitude on the *y*-axis. Axis labels were omitted as they would be too small or too crowded to see, but the scaling is identical to that in Figure 5B, i.e., the abscissas cover a range of [−5,3] octaves re 500 Hz, and the ordinates cover the range [−0.1 × y_max, y_max], where y_max is the maximum response observed across all stimulus types and recording sites in the corresponding multielectrode penetration. The curves are shown with error bars (mean + standard error) but error bars are very small and may be hard to see. Tuning curves from recording sites inside the ICc are plotted against a white background, those outside the ICc are shown against a light gray background.

The majority of multiunits in our data set exhibited a strong dependence of periodicity tuning on stimulus type, as can be seen in Figure 6, which shows rMTFs for all recording sites from two representative penetrations. Total response (corresponding to the area under the curves in Figure 5) tuning curves are plotted in blue for click trains, green for SAMN and red for IRN. The responses are normalized relative to the maximum observed in the respective mulitelectrode penetration. A green square marks the recordings which were shown in more detail in Figure 5.

The click train, SAMN, and IRN rMTFs differ substantially in all cases shown. Sometimes the click train rMTFs correlate better with those seen with IRNs, sometimes with those recorded with SAMN, and sometimes there is little discernible relationship between any of them. The sample of rMTFs shown in Figure 6 thus illustrates some of the diversity of response properties that can be observed in the ICc. Particularly near the edges of the ICc, responses to the periodic stimuli may be weak or nonexistent and show no apparent preference for any particular MF values. Near the center of the ICc, click trains rMTFs often feature a prominent, broad peak for MFs near 500 Hz, and possible additional sharp peaks for MFs in excess of 1000 Hz. In contrast, responses to SAMN and IRN were usually, but not always, weaker than those to click trains (for both penetrations, the absolute strongest response seen was evoked by SAMN stimuli). Note that all rMTFs in Figure 6 are plotted with standard error of mean error bars, but since responses to individual stimuli tended to be highly reproducible and the number of repeat presentations in the sample (at least 25 for each stimulus) was relatively large, the error bars are often too small to see. The trends and the multiple local maxima and minima which are apparent in many of the tuning curves shown are therefore not “noise”, but are a statistically robust reflection of the complexities of the underlying neural response preferences.

### Attempting to Capture Modulation Tuning with a Single Measure, Such as the Best Modulation Frequency, is Problematic

The diversity and complexity of rMTFs illustrated in Figure 6 reveals some of the problems associated with trying to summarize the dependence of the response on MF by citing only the BMF which evoked the maximal response (rBMF). Some tuning curves are largely flat, some have several local maxima which may be several octaves apart, while others appear to rise or fall very gently and more or less monotonically throughout the whole eight octave range tested, so that the observed maximum may only represent a lower or upper bound on the true rBMF. While some studies have grappled with the diversity of rMTFs (Krishna and Semple, 2000), many previous studies focus solely or entirely on the anatomical and the statistical distributions of rBMFs (Langner and Schreiner, 1988; Schreiner and Langner, 1988; Miller et al., 2002; Baumann et al., 2011). To facilitate comparison with that previous body of work, we report rBMFs in the following paragraphs and figures, but include only rBMF values from those multiunits which showed a “robust” dependency of response on stimulus MF, in the sense that changes in MF could account for at least 10% of the trial-to-trial response variance. This inclusion criterion ensured that, for all multiunits in the data set, changing MF had an effect on the response which was “sizable”, as well as highly statistically significant (*p* < 10^−22^, one-way ANOVA). Out of the 1022 ICc recordings collected, the number of multiunits that met this inclusion criterion was 904 for click trains, 648 for SAMN and 600 for IRN, and for each, the rBMF was determined as that MF which evoked the maximal response, in keeping with common practice.

### Unlike Best Frequencies, Best Modulation Frequencies do not Form Clear and Consistent Anatomical Gradients Across the ICc

**Figure 7:**
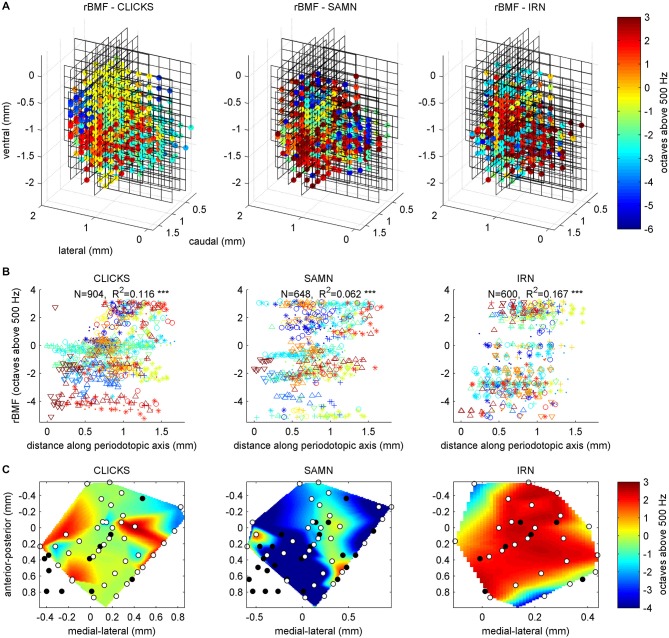
**(A)** Best modulation frequencies shown as 3D color maps (top row) and as scatter plots of response best modulation frequency (rBMF; the “best” modulation frequency which evoked the maximal response) against distance along the best fit periodotopic axis (bottom row). Data are shown for click train (left), SAMN (middle) and IRN (right) stimuli. The 3D maps are laid out as in Figure 4A, with the axes showing the anatomical coordinates of the recording sites. Each black grid shows the position of one multi-electrode array penetration. The colored dots indicate the location of multiunits that were located within the ICc, and for which changes in stimulus MF accounted for at least 10% of the response variance. The color indicates the MF of the stimulus that evoked the strongest response (compare color scale on the right). **(B)** Scatter plots laid out as in Figure 4B, with different colored symbols used to plot the data recorded from each animal. A random Gaussian distributed jitter with a standard deviation of 0.09 octaves has been added to the rBMF values to spread out overlapping data points. The number of multiunits in each data set (*N*) and correlation coefficient (*R*) are shown above each panel. Triple asterisks (*) indicate that the *R* values are statistically significant at *p* < 0.001. **(C)** Periodotopic maps constructed from multiunits within a functionally defined iso-frequency lamina (BFs within 15% of 1 kHz). The ordinate and abscissa give anatomical distances in mm within the iso-frequency plane. The black dots show the positions of multiunits that were excluded from the analysis because their responses were not strongly influenced by stimulus MF. The white dots show the location of multiunits whose rBMFs were used to construct the periodotopic maps by 2-D interpolation.

If, in a first instance, we assume that the nature of the periodotopic map of the gerbil IC might resemble that of the tonopic map, with clear gradients running throughout the nucleus, in a direction that is consistent from animal to animal, then visualizing and analyzing the rBMF distributions in the same way as we had done for the tonotopy in Figure 4 should give a good overview of the periodotopic order. We therefore show the anatomical distribution of rBMFs for the click train (left) SAMN (middle) and IRN (right) stimuli respectively in Figure 7A.

Two observations are immediately apparent. Firstly, for all three stimulus types tested, there is no very obvious and consistent periodotopic gradient which spans the entire length, depth or width of the nucleus and which would be comparable in clarity to the tonotopic gradient shown in Figure 4. Secondly, the rBMF maps obtained with click-trains, SAMN or IRN stimuli respectively look very different. In other words, in the gerbil ICc, periodotopy is clearly not invariant with respect to stimulus type.

### Best Fit Periodotopic Gradients Leave Most of the Periodotopic Structure Unexplained and do not Show a Consistent Orientation Across Stimulus Types

To quantify these observations we fitted periodotopic gradient models to the rBMF data, using the same linear regression methods as those used to quantify the tonotopy in the context of Figure 4 above. Table 1 compares the results of the regression analyses with either BF or with the rBMFs obtained with each of the three periodic stimuli as dependent variables. The table shows that, for all stimulus types, even though there are no obvious periodotopic gradients in the figures, the regression analyses nevertheless return statistically significant associations between rBMF and anatomical coordinates. However, it is important to bear in mind that the small *p*-values shown in Table 1 merely indicate that a null hypothesis which assumes the distribution of rBMFs to be completely random and independent of anatomical location provides an even worse description of the data than an alternative hypothesis which assumes rBMF to depend linearly on anatomical location. It does not imply that a linear anatomical gradient is necessarily the most appropriate (or even a good) description of the periodotopic organization. Indeed, the low proportion of the variance in rBMF that is explained by the regression indicates that the data are quite poorly described by a linear periodotopic gradient model. While the best fit tonotopic gradient explains 69% of the variance in BF, the periodotopic gradients explain only between 6 and 17% of the variance in rBMF.

**Table 1:** **Comparison of regression analyses quantifying the tonotopy shown in Figure 4 and the periodotopies shown in Figure 7A**.

	*N*	Variance explained	*p* value	b_ML	b_DV	b_RC
Tonotopy	1022	0.690	1.00E-120	−2.03	3.07	1.78
Periodotopy, click trains	904	0.116	7.08E-024	−0.44	0.49	1.73
Periodotopy, SAMN	648	0.062	5.38E-009	0.25	1.55	−2.36
Periodotopy, IRN	600	0.167	1.63E-023	−2.45	2.45	−0.40

*The number of data points in the regression, the proportion of the variance explained by the regression, and its statistical significance are shown along with the regression weights b_ML, b_DV and b_RC, which indicate the change in BF or rBMF in octaves predicted by the linear topographic gradient model for every mm moved in a lateral, ventral or caudal direction respectively. Note that data from all animals were pooled for these analyses. As discussed further in the text, while the tonotopic organization is very similar from animal to animal, the periodotopic organization does exhibit considerable individual variability*.

We also note that the “best fit periodotopic axes” for the different types of periodic stimuli are very poorly aligned. As discussed above, the regression weights b_ML, b_DV and b_RC shown in Table 1 represent the *x*, *z* and *y* coordinates of a vector pointing in the direction of the corresponding periodotopic axis. From these we can calculate that the angle between the best fit periodotopic axes for the IRN data and the SAMN data is as large as 56°. Also, the predicted rate at which best modulation frequencies are expected to change as one moves along the best fit periodotopic axis is more than twice as large for IRN as for SAMN (3.5 vs. 1.7 octaves/mm respectively). Scatter plots showing the distribution of rMBFs around the best fit periodotopic axes are shown in Figure 7B. Comparison with Figure 4B reveals important differences between the tonotopic and the periodotopic organization of IC. Not only is the scatter in Figure 4B generally smaller than that seen in Figure 7B, we also note that datapoints from individual animals do not tend to cluster in Figure 4B, as they clearly do in Figure 7B, which hints at the possibility that the periodotopic organization, unlike the tonotopic map, may exhibit substantial individual differences. This is indeed the case, as will be discussed in much greater detail below, but first we shall further investigate the mutual relationships between BF and rBMF in a manner that does not depend on the question whether ICc periodotopy is consistent from animal to animal or not.

### Periodotopic Gradients are not Anatomically Orthogonal to Tonotopic Gradients

Previous work (Schreiner and Langner, 1988; Baumann et al., 2011) had also led to the hypothesis that tonotopic and periodotopic representations in the ICc may be orthogonal to each other. The technical term “orthogonal” can take slightly different meanings in different contexts, but one interpretation is that tonotopic and periodotopic gradients are anatomically at right angles to each other. Schreiner and Langner (1988) suspected such an arrangement, and therefore assumed that sampling ICc multiunits deliberately along functionally defined iso-frequency laminae (i.e., collecting only multi-units with similar BFs) might reveal any underlying periodotopic gradients particularly clearly. Note, however, that such iso-BF sampling should not be strictly necessary, and indeed Baumann et al. (2011) report seeing periodotopic gradients in their imaging data even though their imaging planes did not run parallel to iso-frequency laminae. Note also that our data do not suggest that “best fit” periodotopic gradients obtained from the pooled data would be orthogonal to the tonotopic gradient, and therefore be aligned with iso-frequency laminae: using the regression weights shown in Table 1 to calculate the angles between the tonotopic and each of the best fit periodotopic axes, we find these to come out as 43.7° for the click train stimuli, 51.2° for SAMN, and 45.9° for IRN. In other words, in none of the cases did the best fit periodotopic axis run even approximately orthogonal to the tonotopic axis. Nevertheless one might ask whether our data would reveal clearer periodotopic gradients if, like Schreiner and Langner (1988), we were to focus on an individual frequency lamina, using data from just one individual animal so that differences between animals do not complicate the picture. Although our sampling method is not optimized for producing numerous periodotopic maps within such individual iso-frequency laminae, our datasets are nevertheless large enough to allow us to extract some representative examples, one of which is shown in Figure 7C. It shows periodotopic maps for click trains, SAM noise and IRN constructed from 63 multiunits from one single animal, all of which had BFs of *ca*. 1 kHz (± 15%). The periodotopic organization shown by these maps is, again, marked by clustering of rBMFs, poorly accounted for by a periodotopic gradient, and strongly dependent on stimulus type.

### BMFs are, However, “Nearly Statistically Orthogonal” to BFs and to Each Other

**Figure 8:**
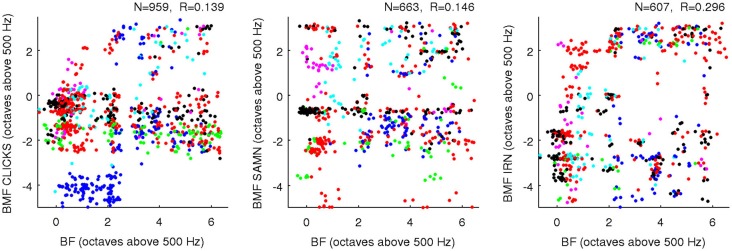
**Scatter plots comparing BF (*x*-axis) against rBMF obtained for each multiunit with each of the three types of periodic stimuli (as indicated on the axes)**. Data from different animals are plotted using dots of different colors, and a random Gaussian distributed jitter with a standard deviation of 0.09 octaves has been added to the rBMF values to spread out overlapping data points. The number of multiunits in each data set (*N*) and correlation coefficient (*R*) are shown above each panel.

Our data thus argue strongly against the idea that the gerbil ICc might exhibit a periodotopic gradient which runs anatomically orthogonal to the tonotopic axis. However, the term orthogonal can also be used more abstractly to describe a lack of correlation or a linear independence between pairs of random variables, and we also examined whether rBMFs and BFs were orthogonal in this sense. Figure 8 shows scatter plots of BF against rBMF for all multiunits and for each stimulus type. The correlations between rBMF and BF in each case are quite small, but given the large N in these data sets, even these small correlations would reach statistical significance if analyzed with the standard *t*-test statistic for assessing the significance of correlations which is, for example, built into the Matlab function *corrcoef()*. However, it is important to bear in mind that these significance tests are predicated on the assumption that the samples in the dataset are statistically independent samples when conditioned on the variables under study (here BF and rBMF). This conditional independence assumption is not safe for the data shown in Figure 8, as statistical dependencies that are likely to result for example from the fact that some values come from neighboring recording sites while others come from sites far apart are not taken into account. It is therefore not straight forward to assess the statistical significance of the observed correlations between BF and rBMF, but it is also not very important because small correlations are unlikely to have much functional significance. Indeed, the observed scatter in Figure 8 is so large that it covers essentially the entire range of pure tone frequency and MF combinations tested about as evenly as one might expect given our sample size and sampling methodology. BF and rBMF can be thus be considered “nearly orthogonal” statistically, and the functional implication of this is that neurons in the ICc appear to cover a full range of rBMFs in each frequency channel.

**Figure 9:**
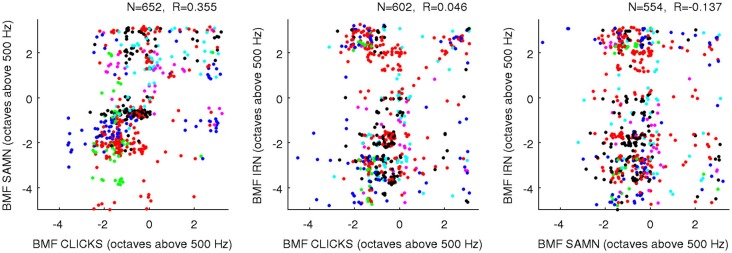
**Scatter plots comparing the rBMFs obtained for each multiunit with each of the three types of periodic stimuli (as indicated on the axes)**. As in Figure 8, data from different animals are plotted using dots of different colors to show that the distribution of rBMFs was comparable across animals; a random Gaussian distributed jitter with a standard deviation of 0.09 octaves has been added to the rBMF values to spread out overlapping data points. The number of multiunits in each data set (*N*) and correlation coefficient (*R*) are again shown above each panel.

One “upside” of the fact that BFs and rBMFs are nearly orthogonal in the statistical sense but not the anatomical sense is that, even though rBMFs are often very different for different stimulus types, these parameters can all be statistically nearly orthogonal both with BF and with each other without running the risk of running out of anatomical dimensions. That the rBMFs observed with each of the three types of periodic stimuli are indeed very different is illustrated in Figure 9, which shows pairwise comparisons of rBMFs across stimuli for each multiunit in the data set as scatter plots. The correlations between rBMFs obtained with the different stimuli are also perhaps smaller than one might expect. While BMFs for clicks and SAMN do show a “reasonable” degree of correlation at *R* = 0.35, IRN BMFs clearly correlate poorly with those observed with either click trains or SAMN.

### Most of the Periodotopic Structure is Attributable to Local Clustering which Differs from Animal to Animal

We have seen that, although rBMF values vary considerably depending on the type of periodic stimulus used, and are not well predicted by a periodotopic gradient running through the ICc, the anatomical distributions of rBMFs are nonetheless clearly not completely random. Indeed, the plots in Figure 7 suggest that rBMFs occur in local clusters which may be somewhere between 0.4 and 1 mm wide. The likely basis for this apparent “periodotopic clustering” is observable in Figure 6, where one can see that periodicity tuning curves recorded in adjacent recording sites are often quite similar. This suggests that the periodotopic organization of ICc may be rather different in nature than hitherto proposed. Indeed, periodotopic maps in the ICc may be more similar to orientation tuning maps in visual cortex rather than to the tonotopic gradient maps found in many central auditory structures. In contrast to tonotopic maps, which present a clear single gradient that spans an entire anatomical structure and which vary little from one individual to the next, visual cortex orientation tuning maps have little or no global gradient but a strong local organization, and the precise structure of the map normally varies considerably from one animal to the next, presumably because this type of map is shaped more by somewhat stochastic, post-natal, activity dependent influences than by genetically predetermined mechanisms (Chapman and Stryker, 1993; Sengpiel et al., 1999; White et al., 2001).

Our data do not speak directly to the relative importance of genetically predetermined or activity driven influences in shaping the periodotopic organization in the ICc, but they do allow us to examine the local periodotopic structure quantitatively and to ask whether it is reproducible or variable from animal to animal. Any periodotopic mapping, whether globally or locally structured, implies that the periodicity tuning curves recorded at two sites in close proximity from each other should be on average, a lot more similar than the tuning curves from a pair of recordings sites picked randomly regardless of their anatomical distance. However, if the periodotopic structure varies from animal to animal, then this “excess similarity” among recordings from nearby anatomical coordinates will be less evident if we compare pairs of tuning curves recorded in different animals.

**Figure 10:**
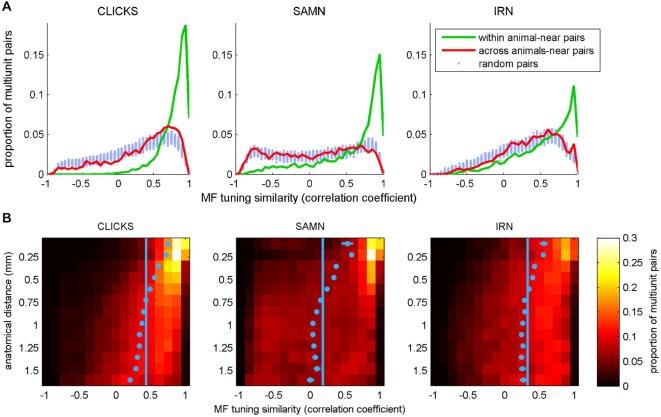
**(A)** Histograms plotting the distribution of correlation coefficients between pairs of periodicity tuning curves, where pairs were chosen either randomly (“random pairs”, light blue markers) or from recording sites with anatomical coordinates that were no more than 0.3 mm apart (“near pairs”). The two tuning curves in the “near” pairs came from nearby coordinates either in a single animal (“within animal”, green lines) or from two different animals (“across animals”, red curves). **(B)** 2-D histogram illustrating the dependence of the similarity (correlation) between periodicity tuning curves on anatomical distance. In the heat maps, each row shows the distribution of correlation coefficients for pairs of tuning curves recorded at points separated by an anatomical distance within 1/16 of a mm from the bin center shown on the *y*-axis. For small anatomical distances, large positive correlation coefficients predominate (i.e., tuning properties are very similar for the large majority of nearby pairs recording sites). This positive skew declines with increasing anatomical distance. The light blue dots show the mean for each histogram. Their error bars cover a range of ±3.3 SEM. Given the large *N*, some of the error bars may be too small to see. The light blue vertical lines demarcate the overall mean correlation coefficient across all sample pairs at all distances.

To test these predictions, we searched our data sets of periodicity tuning curves for pairs of recordings taken at anatomical coordinates not more than 0.3 mm apart, either within the same animal or in two different animals. The number of distinct “within animal–near pairs” in our data set was 6326 for the click train stimuli, 3957 pairs for SAMN, and 3994 for IRN. The number of “across animal-near pairs” was 10,256 for click trains, 6368 for SAMN and 5704 for IRN. To score the similarity of the rMTFs in each pair we computed their correlation coefficient. The distributions of the correlation coefficients found are shown in Figure 10A in histogram form. To be able to compare these observed distributions against the distributions that might be expected under the null hypothesis that anatomical proximity is irrelevant, we estimated this “null distribution” by drawing 100 sets of 10,000 randomly selected pairs each, computed their correlation coefficients and plotted their distributions in Figure 10A with pale blue dots. The pale blue dots thus occupy an approximate 99% confidence interval for the null hypothesis that rMTF similarity is independent of anatomical proximity. For all stimulus types tested, the distribution for the within animal-near pairs (green line) exhibits a much greater skew toward large positive correlation coefficients than that seen in the null distributions, indicating that rMTFs from nearby recording sites within the same animal are clearly on average much more similar than would be expected by chance. In contrast, the distributions obtained from the “across animal-near pairs” are not very different from the null distributions, which indicates that the periodotopic structure is not the same from animal to animal.

### Tonotopic Gradients are Highly Reproducible from One Animal to Another. Periodotopic Gradients are not

Another test to ask whether the periodotopic organization of ICc varies from animal to animal is to ask whether linear regression analysis to determine best fit periodotopic axes for different animals would give consistent results. However, this test is reliable only if the data sets from each animal included in the test comprise large samples covering a wide and substantially overlapping range of anatomical coordinates. Data sets from two of our animals, with sample sizes of 350 and 192 multiunits respectively, meet this criterion. We found the best fit periodotopic axes for these two animals to be poorly aligned, running respectively at angles of 94° to each other for click trains, 64° for SAMN, and 63° for IRN. In contrast, the best fit tonotopic axes for the same two animals are very well aligned, running nearly parallel with an angle of only 4° between them. In the context of Figure 7 and Table 1 above, we had noted that periodotopic gradient maps account for only about 10% on average of the observed variability in rBMF, but given the clear evidence we have just seen that periodotopic maps, unlike tonotopic ones, differ markedly from one animal to the next, one might of course ask whether periodotopic gradient maps fitted to each animal individually provide a better fit. Maybe periodotopic gradient maps are a good model if one allows each animal to have its own periodotopic axis. However, if we perform a regression analysis solely on the 350 locations sampled from the animal in which we obtained the largest dataset, we obtain % variance explained values quite similar to those seen for the pooled data in Table 1, i.e., for this animal, anatomical location relative to its own individual best fit periodotopic axes accounts for only about 11% of the variance in rBMF for click trains, 7% for SAMN and 20% for IRN. In contrast, for that same animal, the best fit tonotopic map accounts for as much as 58% of the variance in BF. This is a key finding: while tonotopic gradients account for most of the variance in frequency tuning in the IC, periodotopic gradients leave almost all of the variability in periodicity tuning from one recording site to another unexplained. Their “statistical significance” notwithstanding, linear periodotopic gradient maps thus do a very poor job at capturing the anatomical distribution of rBMFs, even if possible individual differences in the directions of periodotopic gradients are allowed for.

### Periodotopic Clustering has a Spatial Scale of Approximately 0.5 mm

In addition to the data in Figure 10A, these observations strongly support the notion that the periodotopic organization in ICc is locally structured and individual to each animal, but they do not give a sense of the “spatial scale” of this localized periodotopic clustering. To appreciate how rapidly the similarity of rMTFs declines with increasing anatomical distance, we binned all the within-animal pairs in our data set (N = 99,416 for click trains, 53,897 for SAMN and 47,992 for IRN) according to anatomical distance into 0.125 mm wide bins and plotted the distribution of similarity scores (correlation coefficients) for each distance bin to generate the 2-D histograms shown as heat maps in Figure 10B. For all three stimulus types, similarity scores are clearly skewed toward large positive values for anatomical distances smaller than about 0.5 mm, but this positive skew shrinks or disappears altogether, for anatomical distances larger than about 0.75 mm. The pale blue markers in Figure 10B show the mean similarity score observed at each anatomical distance. These markers are shown with error bars that extend 3.3 standard errors to either side of the sample mean, and thus provide a rough 99.9% confidence interval for the “true mean”, which can be compared against the overall mean similarity score across all anatomical distances, shown by the pale blue vertical lines. If the blue markers and their error bars are to the right of the vertical blue lines, then the mean similarity score at the corresponding anatomical distance can be considered statistically significantly larger than would be expected under the null hypothesis that tuning similarity does not depend on anatomical distance. The blue markers lie to the right of that line for distributions obtained at anatomical distances less than about 0.5–0.6 mm, so this can serve as an estimate of the “spatial scale” of the ICc’s local periodotopic organization.

### Phase Locking is Predominantly Low-Pass, and also Exhibits Substantial Differences Depending on Stimulus Type

**Figure 11:**
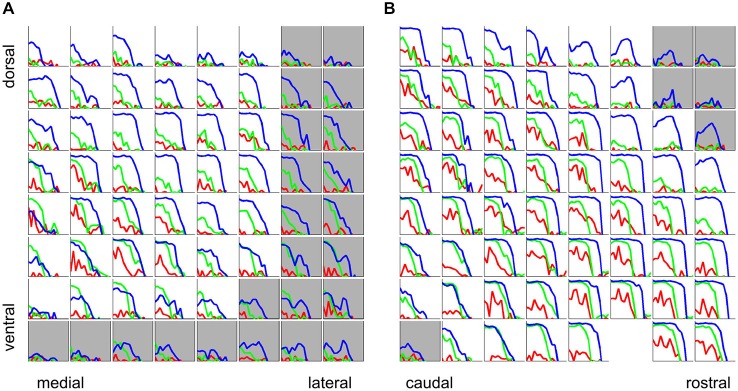
**Phase-locking to the stimulus period for click trains (blue), SAMN (green), and IRN (red) for the same two sample electrode penetrations for which rate tuning curves were shown in Figure 6, one oriented medio-laterally (A), the other oriented rostro-caudally (B)**. Each colored curve shows the phase-locking coefficient as a function of stimulus MF. Tuning curves from recording sites inside the ICc are plotted against a white background, those outside the ICc are shown against a light gray background. Axis labels were omitted as they would be too small or too crowded to see, but all axes are scaled identically to Figure 5C, i.e., the abscissas cover a range of [−4,1] octaves re 500 Hz and the ordinates cover a range of [0, 1].

So far we have considered periodicity tuning mostly in terms of overall response strength, but numerous previous studies indicate that temporal discharge patterns, and in particular, the “phase-locking” of firing to the stimulus period, may also play an important role in encoding stimulus MF, particularly in lower stages of the auditory pathway. The response patterns shown in Figures 5A and C indicate that, particularly for click trains and SAMN, repeated stimulus cycles lead to repeated response patterns, i.e., the discharge pattern phase-locks to the stimulus period. The responses shown in Figure 5 exhibit a number of features that are quite common in our data set, namely, that phase-locking is generally not as strong in the SAMN and IRN as in the click trains, and that the strength of phase-locking declines as stimulus MF rises above a few hundred Hz. In other words, the tMTFs of the multiunit responses we observed in the ICc are mostly low-pass. Figure 11 shows the tMTFs computed, as for Figure 5C, by calculating the mean correlation of the response patterns for successive periods of the stimulus, from the responses recorded on the same two penetrations for which rate tuning to the periodic stimuli was shown in Figure 6 above. This wider sample confirms that: (a) phase-locking to click trains is usually, but not always, stronger than that to SAMN; (b) that phase-locking to IRN is generally weaker and more variable; and (c) that the overwhelming majority of tMTFs are low-pass.

**Figure 12:**
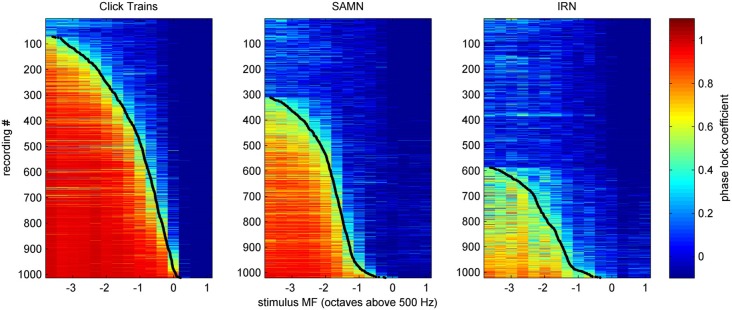
**Phase-locking curves for all multiunit recordings in our sample, in response to click-trains (left), SAMN (center) or IRN (right), sorted by low-pass cutoff frequency**. Each row shows the phase-lock coefficient according to the color scale on the right as a function of stimulus MF (*x*-axis) for one multiunit. Low-pass cutoff frequencies were defined as the stimulus MF for which the phase-lock coefficient dips below a value of 0.4, and these are shown as black dots for each phase-lock tuning curve.

We determined the phase-locking limit (low-pass cutoff) for each multiunit as that value of MF for which the tMTF dipped below a value of 0.4. To estimate each multiunit’s cutoff MF, we smoothed the tMTFs with a 3 point running average filter and interpolated between the two closest MF values tested. Figure 12 shows the tMTFs for all multiunits in our data set, sorted by cutoff MF. The cutoff value of 0.4 to quantify phase locking limits was chosen by inspection of the data shown in Figure 12. The precise choice of cut-off value is not critical. Figure 12 facilitates the comparison of temporal coding of stimulus periodicity in ICc responses for the different stimulus types and makes it easy to read off descriptive statistics regarding the temporal encoding provided by the ICc at a glance. For example, one can easily see that there is essentially no robust phase-locking to stimulus periodicities above 500 Hz in the ICc for any of the stimulus classes tested. Furthermore, for click trains, approximately half the multiunits phase-lock to MFs between about 200 and 400 Hz, and less than 10% appear not to phase-lock to the stimulus period at any MF, while for IRNs, more than half of the multiunits do not phase-lock at all, and very few phase-lock above 200 Hz. Given the weak envelope modulation of IRNs remarked upon in the context of Figure 2 in the “Materials and Methods” Section, this is not a surprising result.

### Phase Locking Shows no Strong or Consistent Periodotopic Gradients Either, but Exhibits Anatomical Clustering

**Figure 13:**
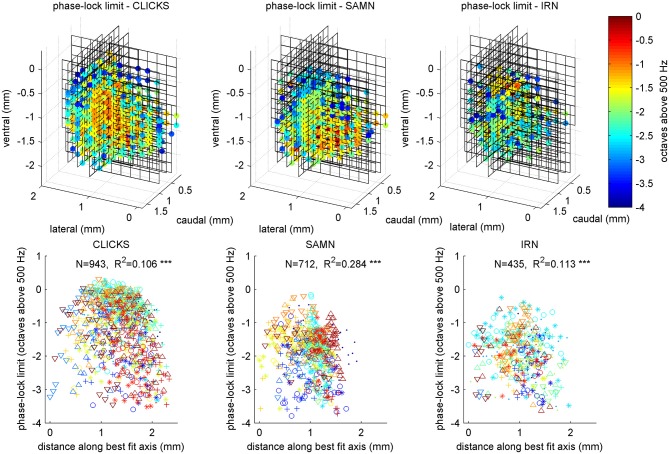
**Anatomical distribution of phase-lock limits (low-pass cutoff MFs) shown as 3D color maps (top row) and as scatter plots of cutoff MF against distance along a best fit anatomical axis (bottom row)**. The layout of the figure is analogous to that of Figure 7. Triple asterisks (*) indicate that the R values are statistically significant at *p* < 0.001.

Figure 13 illustrates the anatomical distribution of periodicity phase-locking limits within ICc. The layout of the figure mirrors that of Figure 7 in which the anatomical distribution of rBMFs was shown. We analyzed these anatomical distributions using linear regression of the phase-locking limits on spatial coordinates, in a manner entirely analogous to the analyses of the tonotopy and rBMF data described above. The results of these analyses are summarized in Table 2. Although the linear regressions have highly significant p-values, phase-locking limits, much like rBMFs, are nevertheless not well captured by a linear periodotopic gradient model. The models explain only modest amounts of the observed variance and the “best fit periodotopic axes” for the three stimulus types are not well aligned (they subtend angles between 25° and 45°).

**Table 2:** **Comparison of results of regression analysis to quantify putative periodotopic gradients in the phase-locking limits illustrated in Figure 13**.

	*N*	Variance explained	*p* value	b_ML	b_DV	b_RC
Tonotopy	1022	0.690	1.00E-120	−2.03	3.07	1.78
Periodotopy, click trains	943	0.106	1.01E-022	0.53	−0.37	0.27
Periodotopy, SAMN	712	0.284	1.00E-120	0.84	−0.18	−0.14
Periodotopy, IRN	435	0.113	3.83E-011	0.11	−0.44	0.69

*For comparison, the values from the analysis of the tonotopy illustrated in Figure 4 are also copied into this table. The number of data points in the regression, the proportion of the variance explained by the regression and its statistical significance are shown, along with the regression weights b_ML, b_DV and b_RC, which indicate the change in BF or cutoff MF respectively in octaves predicted by the linear topographic gradient model for every mm moved in a lateral, ventral or caudal direction respectively*.

**Figure 14:**
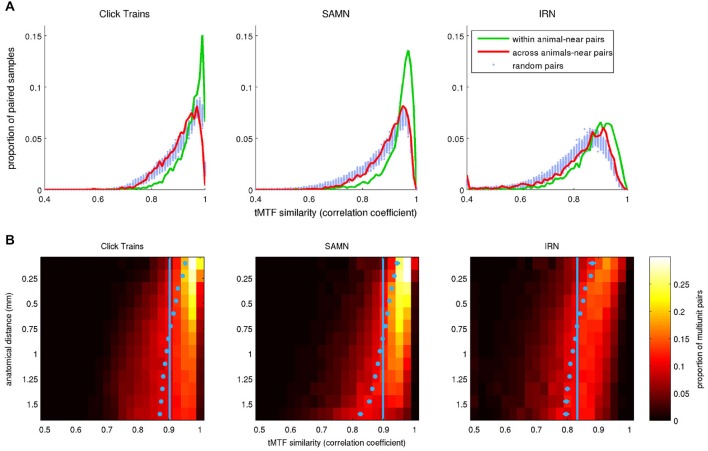
**(A)** Histograms plotting the distribution of correlation coefficients between pairs of tMTFs, where pairs were chosen either randomly (“random pairs”, light blue markers) or from recording sites with anatomical coordinates that were no more than 0.3 mm apart (“near pairs”). The two tuning curves in the “near” pairs came from nearby coordinates either in a single animal (“within animal”, green lines) or from two different animals (“across animals”, red curves). **(B)** 2-D histogram illustrating the dependence of the similarity (correlation) between tMTFs on anatomical distance. In the heat maps, each row shows the distribution of correlation coefficients for pairs of tuning curves recorded at points separated by an anatomical distance within 1/16 of a mm from the bin center shown on the *y*-axis. The light blue dots show the mean for each histogram. Their error bars cover a range of ±3.3 SEM. Given the large *N*, some of the error bars may be too small to see. The light blue vertical lines demarcate the overall mean correlation coefficient across all sample pairs at all distances.

To examine whether, like rBMFs, the anatomical distribution of phase-locking limits also exhibited local clustering, we analyzed the similarity between tMTFs as a function of the distance between the sites from which they were recorded. As shown in Figure 14, tMTFs were generally more similar across the IC than rMTFs (compare to Figure 10), but the extent of the local clustering of tMTFs and rMTFs were similar, suggesting that rate- and timing-based periodicity tuning in ICc are organized on similar spatial scales.

## Discussion

In this study, we used array electrodes to study the periodotopic organization of the gerbil ICc. By making multiple penetrations with a planar array, we were able to sample responses from a large number of recording sites across three dimensions with ms temporal resolution and spatial resolution of 0.2 mm or better in each animal. This approach enabled us to map responses to a larger set of periodic stimuli and a wider range of MFs than in previous studies, and revealed a picture of the periodotopic organization of the IC of the gerbil that is different to that described for other mammals. Unlike the majority of previous studies, we used several different types of periodic stimuli and showed substantial differences in modulation tuning for the different classes. We also extended the range of MFs tested up to several kHz, and found some multiunits to exhibit rBMFs well above those tested in the majority of previous studies.

While our data revealed a clear tonotopic gradient which accounted for most of the site-to-site variation in BFs, we found that even best fit periodotopic gradients running through the ICc left 80% or more of the variation in BMFs unexplained. Rather than conforming to a global gradient, MF tuning curves instead exhibited a strong local ordering, characterized by anatomical clustering of similar modulation tuning curves on a spatial scale of *ca*. 0.5 mm. These locally organized periodotopic maps differed from one animal to the next, unlike the tonotopic map which was highly conserved between individuals. In this respect our findings differ significantly from previous descriptions of the periodotopic organization of the IC based on either single microelectode recordings in cats (Schreiner and Langner, 1988) and chinchillas (Langner et al., 2002), or fMRI studies in monkeys (Baumann et al., 2011). It is of course possible that there may be genuine species differences in the periodotopic organization of the ICc. For example, recent work suggests that there may be systematic differences between smaller and larger species in the manner in which midbrain neurons are tuned to binaural, spatial cues (Harper et al., 2014), so it might not be implausible to assume that the tuning to stimulus periodicities might also exhibit systematic stimulus differences. However, the results we present here are in good agreement with a study by Müller-Preuss et al. (1994) which studied responses of neurons in the IC of squirel monkeys and also failed to find any evidence for clear periodotopic gradients. It therefore seems unlikely that the different conclusions between our study and that by Baumann et al. (2011) can be attributed to systematic differences between rodents and primates. Rather, we propose that the differences between our findings and those of previous studies stem from the fact that the recording techniques used in these previous studies were not sufficient to reveal the local organization we observed. Neither the use of a single microelectrode (Schreiner and Langner, 1988) nor the use of fMRI (Baumann et al., 2011) produces data sets large enough and of sufficient spatial resolution to reveal that gradient maps can only account for a very small share of the anatomical variation in modulation tuning, and that the anatomical arrangement is much better accounted for by strong, individual and local clustering of response properties.

Indeed, on a more macroscopic level, there are a number of noteworthy similarities between these previous descriptions of IC periodotopy and ours. For example, Schreiner and Langner (1988) describe observing concentrically arranged iso-rBMF contours organized around a “highest-rBMF cluster” centered on the middle lateral third of an iso-frequency lamina. Their paper does not provide enough detail to draw quantitative comparisons of the clustering they describe with the strong local clustering we have observed here, but in many respects their results may be more compatible with ours than they might at first appear. As we have seen in Figure 10, our data clearly indicate strong individual differences in the periodotopic organization from one animal to the next. Schreiner and Langner’s (1988) report makes no such reports of individual differences in the cat, but the question is not addressed directly and it is not clear whether their data set was large enough to examine this question.

In contrast to Schreiner and Langner (1988), who report a concentric arrangement of rBMFs around a localized cluster in the IC of the cat, Baumann et al. (2011) describe the periodotopy of the monkey IC in terms of a linear gradient running orthogonal to the tonotopic axis observed in the best MFs seen in voxels studied with functional MRI. On the other hand, several recent studies have reported that IC cells with low BFs are sensitive to faster modulations than those with high BFs (Middlebrooks and Snyder, 2010; Rodríguez et al., 2010a,b), and these findings are consistent with differences in the membrane time constants of neurons with low and high BFs (Geis and Borst, 2009). These results might suggest the possibility of a periodotopic gradient that runs in parallel, rather than orthogonal to the tonotopic axis. However, parallel tonotopic and periodotopic gradients would imply constraints on the range of periodicities that can be represented in any one frequency band which would be of doubtful ecological usefulness. It is therefore noteworthy that our data indicate that no periodotopic gradient direction, whether parallel or orthogonal or obliquely to the tonotopic axis, is able to account for more than a small fraction of the observed periodotopic structure.

While the local periodotopic clustering we have observed is not well described in terms of a linear gradient map, there were nevertheless statistical dependencies between tuning at different sites across the IC which in turn led to significant correlations between spatial coordinates and rBMFs. Indeed all the linear regression analyses in Tables 1 and 2 above are highly significant, and might be construed as evidence for periodotopic gradients, in qualitiative agreement with the results by Baumann et al. (2011). Only when we study the periodotopic arrangement at a higher spatial resolution does the poor explanatory power of the periodotopic gradients and the lack of consistency across stimulus types and animals become apparent. Our results thus suggest that, rather than the linear periodotopic gradients observed by others, MF tuning is organized by clustering on a local scale. The local clustering of MF tuning that we observed is consistent with the observations of Chen et al. (2012), who used analysis of spectrotemporal receptive fields (STRFs) to demonstrate that neighboring IC cells had similar preferences for envelope modulations. Beyond modulation tuning, local clustering has been observed for some response properties in the IC, but not others. For example, using tetrode recordings, Seshagiri and Delgutte (2007) showed that neighboring IC cells have similar pure-tone thresholds, but not similar FRA types, pure-tone post-stimulus time histogram (PSTH) types, or interaural differences in timing (ITD) tuning.

Another important new observation in this study is that we saw that MF tuning depended greatly on stimulus type. Different types of periodic stimuli produce different tuning curves, and these tuning curves are often poorly summarized by simply citing the observed rBMF. The resulting dependence of periodotopy on stimulus type is in apparent contrast with the tonotopic organization, since previous work has shown neural BF estimates to be very similar irrespective of whether they are measured using pure tones or reverse correlation to broad-band sounds (Escabi and Schreiner, 2002). However, this dependence of modulation tuning on stimulus type is consistent with previous studies which have shown responses of ICc neurons to depend on a wide variety of stimulus features. For example, the responses of neurons in ICc of bats, mice, and gerbils have been shown to vary with the “duty cycle”, that is, the duration of the “on phase” of the periodic stimulus (Casseday et al., 1994; Brand et al., 2000; Fremouw et al., 2005; Krebs et al., 2008) or the envelope shape (Zheng and Escabí, 2008, 2013), even when the overall repetition rate of the stimulus is constant. Changes in modulation tuning have also been observed with changes in more global features of a stimulus including mean level, modulation depth, background noise level, and spatial position (Rees and Palmer, 1989; Koch and Grothe, 2000; Krishna and Semple, 2000). Similarly, neurons in higher centers of the auditory pathway have also been found to exhibit joint sensitivity to stimulus period as well as other parameters such as spectral envelope (Bizley et al., 2009; Walker et al., 2011). Stimulus periodicity is thus clearly only one of many factors shaping responses in ICc, and these factors may well interact non-linearly, changing the shape of the MTF depending on a variety of stimulus attributes. If we further bear in mind that the SAMN and IRN stimuli used here differ greatly in the extent to which they express periodicity in their envelope or fine structure respectively, then the observed differences rMTFs obtained with these different stimulus types should not come as a great surprise.

Recent work has seen the IC become a potential target for neuroprosthetic implantation, with the hope to restore functional hearing in patients with auditory nerve damage (Lenarz et al., 2006). In addition, psychoacoustic studies suggest that different “modulation channels” may play different key roles in conveying important aspects of auditory signals such as speech (Woolley et al., 2005; Elliott and Theunissen, 2009). A more detailed understanding of the periodotopic anatomy of the IC is therefore potentially of considerable clinical interest. The substantial differences in MF tuning from subject to subject and from one stimulus class to another which we have documented here, suggest that the development of sophisticated midbrain implants which might try to target particular modulation channels could face significant difficulties. However, individual differences in the organization of neural maps are often the hallmark of activity dependent processes driving map formation, which allow these maps to exhibit considerable functional plasticity (Sengpiel et al., 1999; White et al., 2001). The substantial individual differences we see in the ICc periodotopic organization thus hint at the interesting, and potentially clinically important, possibility that the representation of periodic sounds in ICc might be very plastic, at least during early development.

## Conflict of Interest Statement

The authors declare that the research was conducted in the absence of any commercial or financial relationships that could be construed as a potential conflict of interest.
